# Chloridobis(1,10-phenanthroline-κ^2^ *N*,*N*′)copper(I) dichloridocopper(II)

**DOI:** 10.1107/S1600536810054073

**Published:** 2011-01-22

**Authors:** Qingfen Meng, Yanmei Chen, Bing Li, Sanping Chen, Shengli Gao

**Affiliations:** a Department of Chemistry, Northwest University, Xi’an 710069, People’s Republic of China; b Qinghai Institute of Salt Lakes, Chinese Academy of Sciences, Xining 810008, People’s Republic of China

## Abstract

The asymmetric unit of the title compound, [CuCl(C_12_H_8_N_2_)_2_]·[CuCl_2_], contains two complex Cu(II) cations and two cuprate(I) anions. The Cu(II) atom is coordinated by two phenanthroline (phen) mol­ecules and one chloride anion in a distorted trigonal–bipyramidal geometry. The Cu(II) complex cations form layers through π–π stacking [interplanar distance = 3.481 (2) Å]. The dichloridocuprate(I) anions are located between the layers, forming a sandwich-like structure.

## Related literature

For the use of 1,10-phenanthroline (phen) in copper complexes, see: Wang *et al.* (2002 ▶, 2003 ▶); Lan *et al.* (2007 ▶). For complexes involving five-coordinated copper atoms with 1,10-phen ligands, see: Gkioni *et al.* (2008 ▶); Mao *et al.* (2004 ▶); Ma *et al.* (2000 ▶); Hu *et al.* (2006 ▶). For mixed-valence copper complexes with 1,10-phen, see: Xu *et al.* (2007 ▶). 
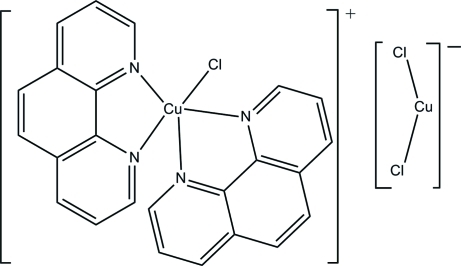


## Experimental

### 

#### Crystal data

[CuCl(C_12_H_8_N_2_)_2_]·[CuCl_2_]
*M* *_r_* = 593.84
Monoclinic, 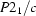

*a* = 9.8137 (3) Å
*b* = 17.8813 (6) Å
*c* = 26.2679 (7) Å
β = 90.535 (1)°
*V* = 4609.3 (2) Å^3^
*Z* = 8
Mo *K*α radiation
μ = 2.21 mm^−1^
*T* = 296 K
0.18 × 0.15 × 0.02 mm

#### Data collection

Bruker APEXII CCD diffractometer
Absorption correction: multi-scan (*SADABS*; Bruker, 2001 ▶) *T*_min_ = 0.691, *T*_max_ = 0.957
35792 measured reflections
9043 independent reflections
6276 reflections with *I* > 2σ(*I*)
*R*_int_ = 0.032

#### Refinement

*R*[*F* ^2^ > 2σ(*F* ^2^)] = 0.038
*wR*(*F* ^2^) = 0.090
*S* = 1.01
9043 reflections
595 parameters
16 restraints
H-atom parameters constrained
Δρ_max_ = 0.65 e Å^−3^
Δρ_min_ = −0.55 e Å^−3^

### 

Data collection: *APEX2* (Bruker, 2001 ▶); cell refinement: *SAINT* (Bruker, 2001 ▶); data reduction: *SAINT*; program(s) used to solve structure: *SHELXS97* (Sheldrick, 2008 ▶); program(s) used to refine structure: *SHELXL97* (Sheldrick, 2008 ▶); molecular graphics: *SHELXTL* (Sheldrick, 2008 ▶); software used to prepare material for publication: *SHELXTL*.

## Supplementary Material

Crystal structure: contains datablocks I, global. DOI: 10.1107/S1600536810054073/jh2249sup1.cif

Structure factors: contains datablocks I. DOI: 10.1107/S1600536810054073/jh2249Isup2.hkl

Additional supplementary materials:  crystallographic information; 3D view; checkCIF report

## Figures and Tables

**Table 1:** Selected bond lengths (Å)

Cu1—N3	1.992 (3)
Cu1—N2	1.999 (3)
Cu1—N4	2.104 (3)
Cu1—N1	2.115 (3)
Cu1—Cl1	2.2721 (9)
Cu2—N5	1.983 (3)
Cu2—N8	1.992 (3)
Cu2—N7	2.125 (2)
Cu2—N6	2.162 (3)
Cu2—Cl2	2.2306 (10)
Cu3—Cl3	2.0813 (12)
Cu3—Cl4	2.0936 (11)
Cu4—Cl6	2.0669 (13)
Cu4—Cl5	2.0706 (11)

**Fig. 1.**
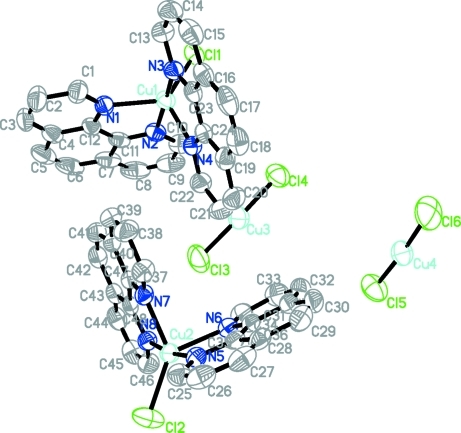
The Molecular structure of 1 showing 50% probability displacement ellipsoids

**Fig. 2.**
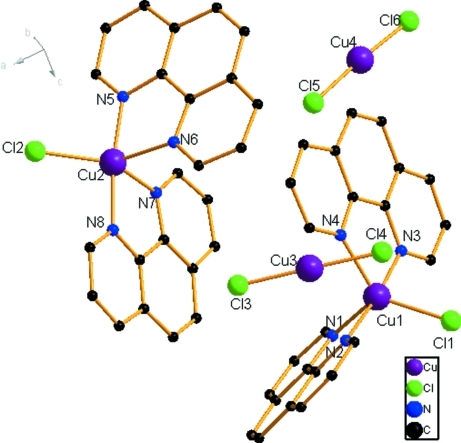
The coordinate environment of complex 1.

**Fig. 3.**
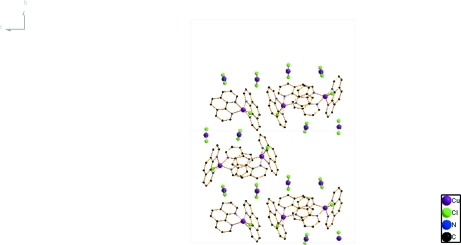
The Packing diagram of 1 viewed down the a-axis.

## supplementary crystallographic information

### Comment

As bidentate diimine ligand, 1,10-phenanthroline (phen), has been widely used as the second ligand in prepare many kinds of metal-coordination complexes, especially in copper(II) complexes (Wang *et al.* 2003, Wang *et al.* 2002, Lan *et al.* 2007). Although many five-coordinated copper(II) complexes are well documented (Gkioni *et al.* 2008, Mao *et al.* 2004, Ma *et al.* 2000, Hu *et al.* 2006), the presence mixed-charge complexes have been rarely observed (Xu *et al.* 2007). Here we report a mixed-charge copper complex with 1,10-phenanthroline as coordinate ligand, this complex was constituted through π-π interactions. The Molecular structure of 1 showing 50% probability displacement ellipsoids was shown in Fig. 1. The coordinate environment of complex 1 shown in figure 2. The Packing diagram of 1 viewed down the *a-*axis was presented in Fig. 3. Selected band length and angle see table 1.

### Experimental

The complex 1 was synthesized by solvothermal reaction. A mixture of 1,10-Phenanthroline monohydrate(0.0198 g, 0.1 mmol), CuCl_2_.2H_2_O (0.0170 g, 0.1 mmol), L1 (L1 = HO-(Ph)—CH=N—Ph—O—Ph—N=CH-(Ph)—OH (0.0204 g, 0.05 mmol) and CH_3_CH_2_OH (3 ml) was sealed in a 6 ml glass tube and heated to 393 K for 72 h. After cooling to room temperature, green crystals were obtained concomitanted with a lot of brown precipitation. The color of solution is light-brown. The ligand of L1 could't coordinate with Cu.

### Refinement

H atoms were placed in calculated positions with C—H = 0.93 (aromatic) refined in riding mode, with *U*_iso_(H) = 1.2 *U*_eq_(C).

### Crystal data

**Table:**

[CuCl(C_12_H_8_N_2_)_2_]·[CuCl_2_]	*F*(000) = 2376
*M**_r_* = 593.84	char
Monoclinic, *P*2_1_/*c*	*D*_x_ = 1.711 Mg m^−^^3^
Hall symbol: -P 2ybc	Mo *K*α radiation, λ = 0.71073 Å
*a* = 9.8137 (3) Å	Cell parameters from 8168 reflections
*b* = 17.8813 (6) Å	θ = 2.2–24.2°
*c* = 26.2679 (7) Å	µ = 2.21 mm^−^^1^
β = 90.535 (1)°	*T* = 296 K
*V* = 4609.3 (2) Å^3^	Rod, green
*Z* = 8	0.18 × 0.15 × 0.02 mm

### Data collection

**Table:**

Bruker APEXII CCD diffractometer	9043 independent reflections
Radiation source: fine-focus sealed tube	6276 reflections with *I* > 2σ(*I*)
graphite	*R*_int_ = 0.032
φ and ω scans	θ_max_ = 26.0°, θ_min_ = 1.4°
Absorption correction: multi-scan (*SADABS*; Bruker, 2001)	*h* = −11→12
*T*_min_ = 0.691, *T*_max_ = 0.957	*k* = −17→22
35792 measured reflections	*l* = −25→32

### Refinement

**Table:**

Refinement on *F*^2^	Primary atom site location: structure-invariant direct methods
Least-squares matrix: full	Secondary atom site location: difference Fourier map
*R*[*F*^2^ > 2σ(*F*^2^)] = 0.038	Hydrogen site location: inferred from neighbouring sites
*wR*(*F*^2^) = 0.090	H-atom parameters constrained
*S* = 1.01	*w* = 1/[σ^2^(*F*_o_^2^) + (0.033*P*)^2^ + 3.6932*P*] where *P* = (*F*_o_^2^ + 2*F*_c_^2^)/3
9043 reflections	(Δ/σ)_max_ = 0.001
595 parameters	Δρ_max_ = 0.65 e Å^−^^3^
16 restraints	Δρ_min_ = −0.55 e Å^−^^3^

### Special details

**Table:**

Geometry. All e.s.d.'s (except the e.s.d. in the dihedral angle between two l.s. planes) are estimated using the full covariance matrix. The cell e.s.d.'s are taken into account individually in the estimation of e.s.d.'s in distances, angles and torsion angles; correlations between e.s.d.'s in cell parameters are only used when they are defined by crystal symmetry. An approximate (isotropic) treatment of cell e.s.d.'s is used for estimating e.s.d.'s involving l.s. planes.
Refinement. Refinement of *F*^2^ against ALL reflections. The weighted *R*-factor *wR* and goodness of fit *S* are based on *F*^2^, conventional *R*-factors *R* are based on *F*, with *F* set to zero for negative *F*^2^. The threshold expression of *F*^2^ > σ(*F*^2^) is used only for calculating *R*-factors(gt) *etc*. and is not relevant to the choice of reflections for refinement. *R*-factors based on *F*^2^ are statistically about twice as large as those based on *F*, and *R*- factors based on ALL data will be even larger.

### Fractional atomic coordinates and isotropic or equivalent isotropic displacement parameters (Å^2^)

**Table:**

	*x*	*y*	*z*	*U*_iso_*/*U*_eq_	
Cu1	0.45874 (4)	0.22779 (2)	0.434313 (15)	0.04415 (11)	
Cl1	0.30626 (8)	0.14861 (5)	0.47027 (3)	0.0552 (2)	
N1	0.6034 (3)	0.28987 (15)	0.47699 (10)	0.0445 (6)	
C1	0.5990 (4)	0.3590 (2)	0.49512 (13)	0.0584 (9)	
H1	0.5211	0.3875	0.4895	0.070*	
Cu2	1.00137 (4)	0.30937 (2)	0.179368 (14)	0.04466 (11)	
Cl2	1.19064 (10)	0.33783 (7)	0.13718 (4)	0.0810 (3)	
N2	0.6088 (3)	0.15237 (15)	0.43836 (10)	0.0462 (7)	
C2	0.7070 (5)	0.3903 (3)	0.52226 (15)	0.0756 (12)	
H2	0.7011	0.4392	0.5340	0.091*	
N3	0.3228 (3)	0.30996 (14)	0.42697 (10)	0.0446 (6)	
C3	0.8191 (5)	0.3503 (3)	0.53145 (14)	0.0751 (13)	
H3	0.8908	0.3714	0.5498	0.090*	
Cu3	0.66098 (5)	0.03206 (3)	0.295513 (19)	0.06514 (14)	
Cl3	0.86153 (12)	0.06908 (8)	0.29074 (5)	0.0969 (4)	
N4	0.4840 (3)	0.25237 (14)	0.35664 (10)	0.0442 (6)	
C4	0.8298 (4)	0.2764 (3)	0.51359 (13)	0.0603 (10)	
Cu4	0.29958 (5)	0.03693 (3)	0.094280 (18)	0.06336 (14)	
Cl4	0.45844 (10)	−0.00298 (6)	0.30242 (4)	0.0770 (3)	
N5	0.8859 (3)	0.37651 (15)	0.13714 (9)	0.0435 (6)	
C5	0.9433 (4)	0.2279 (3)	0.52143 (16)	0.0818 (15)	
H5	1.0179	0.2451	0.5402	0.098*	
Cl5	0.48555 (11)	−0.01769 (6)	0.09709 (5)	0.0866 (3)	
N6	0.8359 (3)	0.23407 (15)	0.16073 (9)	0.0437 (6)	
C6	0.9458 (4)	0.1586 (3)	0.50272 (16)	0.0775 (14)	
H6	1.0219	0.1287	0.5086	0.093*	
Cl6	0.11371 (13)	0.09104 (9)	0.09723 (6)	0.1124 (5)	
N7	0.9086 (2)	0.34430 (14)	0.24817 (9)	0.0421 (6)	
C7	0.8333 (4)	0.1291 (2)	0.47356 (14)	0.0617 (10)	
N8	1.0928 (3)	0.23944 (15)	0.22782 (10)	0.0446 (6)	
C8	0.8270 (4)	0.0571 (3)	0.45286 (16)	0.0758 (13)	
H8	0.8997	0.0245	0.4577	0.091*	
C9	0.7159 (5)	0.0342 (2)	0.42570 (17)	0.0742 (12)	
H9	0.7122	−0.0136	0.4118	0.089*	
C10	0.6071 (4)	0.0841 (2)	0.41908 (14)	0.0600 (10)	
H10	0.5310	0.0687	0.4005	0.072*	
C11	0.7192 (3)	0.1750 (2)	0.46529 (12)	0.0463 (8)	
C12	0.7172 (3)	0.2488 (2)	0.48574 (12)	0.0461 (8)	
C13	0.2456 (3)	0.3388 (2)	0.46311 (14)	0.0572 (9)	
H13	0.2535	0.3199	0.4960	0.069*	
C14	0.1532 (4)	0.3962 (2)	0.45373 (17)	0.0640 (10)	
H14	0.1014	0.4157	0.4801	0.077*	
C15	0.1390 (4)	0.4239 (2)	0.40545 (17)	0.0621 (10)	
H15	0.0774	0.4623	0.3988	0.074*	
C16	0.2169 (3)	0.39434 (18)	0.36624 (14)	0.0492 (8)	
C17	0.2079 (4)	0.4176 (2)	0.31404 (16)	0.0607 (10)	
H17	0.1456	0.4545	0.3048	0.073*	
C18	0.2868 (4)	0.3875 (2)	0.27856 (15)	0.0608 (10)	
H18	0.2770	0.4033	0.2450	0.073*	
C19	0.3862 (3)	0.33160 (19)	0.29055 (13)	0.0507 (9)	
C20	0.4736 (4)	0.2995 (2)	0.25542 (14)	0.0652 (11)	
H20	0.4705	0.3141	0.2215	0.078*	
C21	0.5636 (4)	0.2464 (2)	0.27123 (15)	0.0666 (11)	
H21	0.6231	0.2250	0.2481	0.080*	
C22	0.5671 (3)	0.2238 (2)	0.32202 (13)	0.0542 (9)	
H22	0.6297	0.1875	0.3320	0.065*	
C23	0.3104 (3)	0.33762 (17)	0.37899 (12)	0.0431 (8)	
C24	0.3956 (3)	0.30633 (18)	0.34118 (12)	0.0420 (8)	
C25	0.9155 (4)	0.4463 (2)	0.12439 (13)	0.0544 (9)	
H25	0.9975	0.4669	0.1356	0.065*	
C26	0.8283 (4)	0.4896 (2)	0.09489 (13)	0.0597 (10)	
H26	0.8530	0.5380	0.0858	0.072*	
C27	0.7073 (4)	0.4613 (2)	0.07935 (13)	0.0607 (10)	
H27	0.6484	0.4902	0.0597	0.073*	
C28	0.6706 (3)	0.3883 (2)	0.09285 (12)	0.0497 (8)	
C29	0.5441 (4)	0.3539 (3)	0.07990 (14)	0.0646 (11)	
H29	0.4805	0.3806	0.0607	0.078*	
C30	0.5142 (4)	0.2844 (3)	0.09460 (15)	0.0669 (12)	
H30	0.4295	0.2643	0.0863	0.080*	
C31	0.6104 (3)	0.2402 (2)	0.12295 (13)	0.0522 (9)	
C32	0.5884 (4)	0.1665 (2)	0.13784 (15)	0.0667 (11)	
H32	0.5052	0.1436	0.1309	0.080*	
C33	0.6896 (5)	0.1279 (2)	0.16269 (16)	0.0702 (11)	
H33	0.6762	0.0784	0.1724	0.084*	
C34	0.8129 (4)	0.1636 (2)	0.17332 (13)	0.0567 (9)	
H34	0.8815	0.1368	0.1899	0.068*	
C35	0.7650 (3)	0.34751 (18)	0.12166 (11)	0.0402 (7)	
C36	0.7365 (3)	0.27213 (19)	0.13576 (11)	0.0423 (8)	
C37	0.8161 (3)	0.3964 (2)	0.25723 (13)	0.0541 (9)	
H37	0.7864	0.4267	0.2306	0.065*	
C38	0.7616 (4)	0.4072 (2)	0.30566 (15)	0.0624 (10)	
H38	0.6964	0.4442	0.3108	0.075*	
C39	0.8038 (4)	0.3640 (2)	0.34499 (14)	0.0583 (10)	
H39	0.7675	0.3712	0.3772	0.070*	
C40	0.9017 (3)	0.30851 (19)	0.33726 (12)	0.0459 (8)	
C41	0.9578 (4)	0.2616 (2)	0.37633 (13)	0.0537 (9)	
H41	0.9267	0.2665	0.4095	0.064*	
C42	1.0537 (4)	0.2111 (2)	0.36625 (13)	0.0558 (10)	
H42	1.0886	0.1820	0.3927	0.067*	
C43	1.1044 (3)	0.20054 (18)	0.31602 (13)	0.0459 (8)	
C44	1.2035 (3)	0.1481 (2)	0.30253 (15)	0.0575 (9)	
H44	1.2414	0.1170	0.3272	0.069*	
C45	1.2443 (4)	0.1426 (2)	0.25349 (16)	0.0625 (10)	
H45	1.3101	0.1078	0.2445	0.075*	
C46	1.1868 (3)	0.1895 (2)	0.21661 (14)	0.0564 (9)	
H46	1.2157	0.1854	0.1831	0.068*	
C47	0.9501 (3)	0.30043 (17)	0.28771 (11)	0.0387 (7)	
C48	1.0511 (3)	0.24541 (17)	0.27680 (12)	0.0398 (7)	

### Atomic displacement parameters (Å^2^)

**Table:**

	*U*^11^	*U*^22^	*U*^33^	*U*^12^	*U*^13^	*U*^23^
Cu1	0.0394 (2)	0.0440 (2)	0.0491 (2)	0.00196 (17)	0.00003 (17)	−0.00137 (19)
Cl1	0.0434 (5)	0.0554 (5)	0.0668 (6)	−0.0075 (4)	−0.0009 (4)	0.0041 (4)
N1	0.0449 (16)	0.0486 (18)	0.0398 (15)	−0.0049 (13)	−0.0012 (12)	0.0034 (13)
C1	0.067 (2)	0.057 (2)	0.051 (2)	−0.0112 (19)	−0.0017 (18)	−0.0041 (18)
Cu2	0.0390 (2)	0.0559 (3)	0.0391 (2)	0.00291 (18)	−0.00186 (16)	0.00489 (19)
Cl2	0.0524 (6)	0.1023 (8)	0.0888 (8)	0.0072 (5)	0.0248 (5)	0.0270 (6)
N2	0.0428 (16)	0.0421 (17)	0.0539 (17)	0.0044 (13)	0.0052 (13)	0.0034 (14)
C2	0.084 (3)	0.080 (3)	0.063 (3)	−0.029 (3)	−0.004 (2)	−0.017 (2)
N3	0.0410 (15)	0.0433 (16)	0.0496 (17)	0.0006 (12)	0.0025 (13)	−0.0045 (13)
C3	0.067 (3)	0.113 (4)	0.045 (2)	−0.044 (3)	−0.004 (2)	−0.011 (2)
Cu3	0.0680 (3)	0.0568 (3)	0.0706 (3)	−0.0075 (2)	0.0015 (2)	−0.0070 (2)
Cl3	0.0883 (8)	0.1114 (10)	0.0908 (8)	−0.0490 (7)	−0.0154 (6)	0.0199 (7)
N4	0.0433 (15)	0.0428 (16)	0.0464 (16)	−0.0045 (13)	0.0022 (13)	−0.0046 (13)
C4	0.044 (2)	0.102 (3)	0.0350 (19)	−0.013 (2)	0.0008 (15)	0.014 (2)
Cu4	0.0595 (3)	0.0590 (3)	0.0713 (3)	0.0020 (2)	−0.0088 (2)	0.0022 (2)
Cl4	0.0569 (6)	0.0804 (7)	0.0936 (8)	0.0065 (5)	0.0016 (5)	−0.0047 (6)
N5	0.0455 (16)	0.0484 (17)	0.0364 (15)	−0.0012 (13)	−0.0014 (12)	0.0034 (12)
C5	0.040 (2)	0.151 (5)	0.054 (3)	−0.011 (3)	−0.0011 (18)	0.025 (3)
Cl5	0.0622 (6)	0.0699 (7)	0.1278 (10)	0.0079 (5)	0.0014 (6)	−0.0166 (7)
N6	0.0498 (16)	0.0421 (17)	0.0391 (15)	0.0003 (13)	0.0017 (12)	−0.0002 (13)
C6	0.040 (2)	0.133 (4)	0.059 (3)	0.020 (3)	0.0087 (19)	0.039 (3)
Cl6	0.0822 (8)	0.1347 (12)	0.1196 (11)	0.0435 (8)	−0.0332 (7)	−0.0131 (9)
N7	0.0390 (14)	0.0478 (16)	0.0395 (15)	0.0042 (13)	−0.0061 (11)	−0.0019 (13)
C7	0.049 (2)	0.086 (3)	0.051 (2)	0.016 (2)	0.0162 (18)	0.025 (2)
N8	0.0398 (15)	0.0505 (17)	0.0434 (16)	0.0046 (13)	−0.0029 (12)	0.0003 (13)
C8	0.065 (3)	0.092 (3)	0.072 (3)	0.036 (2)	0.024 (2)	0.029 (2)
C9	0.093 (3)	0.051 (2)	0.079 (3)	0.020 (2)	0.034 (3)	0.007 (2)
C10	0.065 (2)	0.052 (2)	0.064 (2)	0.0036 (19)	0.0131 (19)	0.0041 (19)
C11	0.0366 (18)	0.062 (2)	0.0403 (18)	0.0050 (16)	0.0079 (15)	0.0170 (17)
C12	0.0387 (18)	0.064 (2)	0.0356 (18)	−0.0078 (16)	0.0024 (14)	0.0086 (16)
C13	0.054 (2)	0.061 (2)	0.056 (2)	0.0003 (19)	0.0058 (18)	−0.0081 (19)
C14	0.049 (2)	0.059 (3)	0.084 (3)	0.0069 (19)	0.011 (2)	−0.016 (2)
C15	0.046 (2)	0.044 (2)	0.096 (3)	0.0027 (17)	−0.006 (2)	0.000 (2)
C16	0.0405 (19)	0.0372 (19)	0.070 (2)	−0.0045 (15)	−0.0055 (17)	0.0009 (17)
C17	0.052 (2)	0.045 (2)	0.084 (3)	−0.0096 (17)	−0.021 (2)	0.016 (2)
C18	0.067 (3)	0.056 (2)	0.059 (2)	−0.019 (2)	−0.014 (2)	0.018 (2)
C19	0.055 (2)	0.048 (2)	0.049 (2)	−0.0159 (17)	−0.0045 (17)	0.0062 (17)
C20	0.080 (3)	0.071 (3)	0.045 (2)	−0.021 (2)	0.006 (2)	−0.001 (2)
C21	0.075 (3)	0.070 (3)	0.055 (2)	−0.009 (2)	0.020 (2)	−0.010 (2)
C22	0.054 (2)	0.053 (2)	0.056 (2)	−0.0024 (17)	0.0065 (18)	−0.0055 (18)
C23	0.0390 (17)	0.0384 (19)	0.052 (2)	−0.0093 (14)	−0.0038 (15)	0.0011 (16)
C24	0.0387 (17)	0.0399 (19)	0.047 (2)	−0.0099 (15)	−0.0012 (15)	−0.0028 (15)
C25	0.060 (2)	0.055 (2)	0.048 (2)	−0.0041 (18)	0.0032 (17)	0.0029 (18)
C26	0.082 (3)	0.047 (2)	0.050 (2)	0.009 (2)	0.011 (2)	0.0078 (18)
C27	0.072 (3)	0.067 (3)	0.043 (2)	0.030 (2)	0.0002 (18)	−0.0003 (18)
C28	0.051 (2)	0.060 (2)	0.0385 (18)	0.0173 (18)	−0.0006 (15)	−0.0075 (17)
C29	0.053 (2)	0.086 (3)	0.055 (2)	0.030 (2)	−0.0111 (18)	−0.019 (2)
C30	0.038 (2)	0.099 (4)	0.065 (3)	0.003 (2)	−0.0046 (18)	−0.034 (2)
C31	0.043 (2)	0.065 (3)	0.048 (2)	−0.0019 (18)	0.0044 (16)	−0.0218 (18)
C32	0.057 (2)	0.075 (3)	0.068 (3)	−0.019 (2)	0.011 (2)	−0.028 (2)
C33	0.085 (3)	0.053 (2)	0.073 (3)	−0.019 (2)	0.015 (2)	−0.010 (2)
C34	0.067 (2)	0.055 (2)	0.048 (2)	−0.0010 (19)	0.0045 (18)	−0.0016 (18)
C35	0.0394 (18)	0.051 (2)	0.0298 (16)	0.0074 (15)	0.0006 (13)	−0.0039 (15)
C36	0.0414 (18)	0.053 (2)	0.0322 (17)	0.0035 (16)	0.0033 (14)	−0.0090 (15)
C37	0.055 (2)	0.056 (2)	0.051 (2)	0.0104 (18)	−0.0039 (17)	−0.0012 (17)
C38	0.059 (2)	0.068 (3)	0.060 (2)	0.014 (2)	0.0077 (19)	−0.013 (2)
C39	0.058 (2)	0.071 (3)	0.045 (2)	−0.008 (2)	0.0093 (17)	−0.010 (2)
C40	0.0448 (18)	0.054 (2)	0.0386 (18)	−0.0122 (16)	0.0005 (15)	−0.0025 (16)
C41	0.062 (2)	0.062 (2)	0.0377 (19)	−0.016 (2)	0.0015 (17)	0.0062 (17)
C42	0.061 (2)	0.061 (2)	0.046 (2)	−0.0189 (19)	−0.0127 (18)	0.0187 (18)
C43	0.0428 (18)	0.044 (2)	0.051 (2)	−0.0096 (15)	−0.0097 (15)	0.0075 (16)
C44	0.049 (2)	0.052 (2)	0.070 (3)	−0.0036 (17)	−0.0151 (19)	0.014 (2)
C45	0.050 (2)	0.051 (2)	0.087 (3)	0.0133 (18)	−0.007 (2)	0.001 (2)
C46	0.049 (2)	0.062 (2)	0.058 (2)	0.0097 (18)	0.0015 (17)	−0.0059 (19)
C47	0.0348 (16)	0.0449 (19)	0.0361 (17)	−0.0075 (14)	−0.0035 (13)	−0.0019 (14)
C48	0.0353 (16)	0.0412 (19)	0.0428 (19)	−0.0090 (14)	−0.0060 (14)	0.0026 (15)

### Geometric parameters (Å, °)

**Table:**

Cu1—N3	1.992 (3)	C15—H15	0.9300
Cu1—N2	1.999 (3)	C16—C23	1.406 (4)
Cu1—N4	2.104 (3)	C16—C17	1.435 (5)
Cu1—N1	2.115 (3)	C17—C18	1.331 (5)
Cu1—Cl1	2.2721 (9)	C17—H17	0.9300
N1—C1	1.325 (4)	C18—C19	1.430 (5)
N1—C12	1.354 (4)	C18—H18	0.9300
C1—C2	1.390 (5)	C19—C20	1.390 (5)
C1—H1	0.9300	C19—C24	1.407 (4)
Cu2—N5	1.983 (3)	C20—C21	1.358 (5)
Cu2—N8	1.992 (3)	C20—H20	0.9300
Cu2—N7	2.125 (2)	C21—C22	1.394 (5)
Cu2—N6	2.162 (3)	C21—H21	0.9300
Cu2—Cl2	2.2306 (10)	C22—H22	0.9300
N2—C10	1.321 (4)	C23—C24	1.420 (4)
N2—C11	1.350 (4)	C25—C26	1.386 (5)
C2—C3	1.332 (6)	C25—H25	0.9300
C2—H2	0.9300	C26—C27	1.351 (5)
N3—C13	1.325 (4)	C26—H26	0.9300
N3—C23	1.358 (4)	C27—C28	1.399 (5)
C3—C4	1.406 (6)	C27—H27	0.9300
C3—H3	0.9300	C28—C35	1.396 (4)
Cu3—Cl3	2.0813 (12)	C28—C29	1.424 (5)
Cu3—Cl4	2.0936 (11)	C29—C30	1.335 (6)
N4—C22	1.329 (4)	C29—H29	0.9300
N4—C24	1.357 (4)	C30—C31	1.435 (5)
C4—C12	1.409 (5)	C30—H30	0.9300
C4—C5	1.426 (6)	C31—C32	1.392 (5)
Cu4—Cl6	2.0669 (13)	C31—C36	1.401 (4)
Cu4—Cl5	2.0706 (11)	C32—C33	1.370 (6)
N5—C25	1.326 (4)	C32—H32	0.9300
N5—C35	1.354 (4)	C33—C34	1.393 (5)
C5—C6	1.334 (6)	C33—H33	0.9300
C5—H5	0.9300	C34—H34	0.9300
N6—C34	1.323 (4)	C35—C36	1.426 (4)
N6—C36	1.354 (4)	C37—C38	1.398 (5)
C6—C7	1.438 (6)	C37—H37	0.9300
C6—H6	0.9300	C38—C39	1.352 (5)
N7—C37	1.324 (4)	C38—H38	0.9300
N7—C47	1.361 (4)	C39—C40	1.397 (5)
C7—C8	1.397 (6)	C39—H39	0.9300
C7—C11	1.404 (5)	C40—C47	1.397 (4)
N8—C46	1.319 (4)	C40—C41	1.431 (5)
N8—C48	1.358 (4)	C41—C42	1.333 (5)
C8—C9	1.361 (6)	C41—H41	0.9300
C8—H8	0.9300	C42—C43	1.427 (5)
C9—C10	1.401 (5)	C42—H42	0.9300
C9—H9	0.9300	C43—C44	1.399 (5)
C10—H10	0.9300	C43—C48	1.403 (4)
C11—C12	1.424 (5)	C44—C45	1.356 (5)
C13—C14	1.391 (5)	C44—H44	0.9300
C13—H13	0.9300	C45—C46	1.396 (5)
C14—C15	1.367 (5)	C45—H45	0.9300
C14—H14	0.9300	C46—H46	0.9300
C15—C16	1.393 (5)	C47—C48	1.427 (4)
			
N3—Cu1—N2	174.14 (11)	C18—C17—H17	119.3
N3—Cu1—N4	80.62 (11)	C16—C17—H17	119.3
N2—Cu1—N4	95.68 (11)	C17—C18—C19	121.9 (4)
N3—Cu1—N1	96.30 (11)	C17—C18—H18	119.0
N2—Cu1—N1	80.52 (11)	C19—C18—H18	119.0
N4—Cu1—N1	108.64 (10)	C20—C19—C24	117.3 (3)
N3—Cu1—Cl1	93.26 (8)	C20—C19—C18	124.5 (4)
N2—Cu1—Cl1	92.59 (8)	C24—C19—C18	118.1 (3)
N4—Cu1—Cl1	128.13 (7)	C21—C20—C19	119.3 (4)
N1—Cu1—Cl1	123.23 (7)	C21—C20—H20	120.4
C1—N1—C12	118.3 (3)	C19—C20—H20	120.4
C1—N1—Cu1	131.0 (2)	C20—C21—C22	120.3 (4)
C12—N1—Cu1	110.7 (2)	C20—C21—H21	119.8
N1—C1—C2	122.2 (4)	C22—C21—H21	119.8
N1—C1—H1	118.9	N4—C22—C21	122.2 (4)
C2—C1—H1	118.9	N4—C22—H22	118.9
N5—Cu2—N8	171.56 (10)	C21—C22—H22	118.9
N5—Cu2—N7	92.89 (10)	N3—C23—C16	122.5 (3)
N8—Cu2—N7	80.52 (10)	N3—C23—C24	117.2 (3)
N5—Cu2—N6	79.99 (10)	C16—C23—C24	120.4 (3)
N8—Cu2—N6	94.98 (10)	N4—C24—C19	123.2 (3)
N7—Cu2—N6	92.79 (9)	N4—C24—C23	116.8 (3)
N5—Cu2—Cl2	93.34 (8)	C19—C24—C23	120.1 (3)
N8—Cu2—Cl2	95.07 (8)	N5—C25—C26	122.1 (3)
N7—Cu2—Cl2	136.12 (8)	N5—C25—H25	118.9
N6—Cu2—Cl2	131.05 (7)	C26—C25—H25	118.9
C10—N2—C11	119.0 (3)	C27—C26—C25	119.7 (4)
C10—N2—Cu1	126.6 (3)	C27—C26—H26	120.1
C11—N2—Cu1	114.4 (2)	C25—C26—H26	120.1
C3—C2—C1	120.0 (4)	C26—C27—C28	120.1 (3)
C3—C2—H2	120.0	C26—C27—H27	120.0
C1—C2—H2	120.0	C28—C27—H27	120.0
C13—N3—C23	118.4 (3)	C35—C28—C27	117.0 (3)
C13—N3—Cu1	127.2 (2)	C35—C28—C29	118.4 (3)
C23—N3—Cu1	114.4 (2)	C27—C28—C29	124.6 (3)
C2—C3—C4	120.6 (4)	C30—C29—C28	121.8 (4)
C2—C3—H3	119.7	C30—C29—H29	119.1
C4—C3—H3	119.7	C28—C29—H29	119.1
Cl3—Cu3—Cl4	178.13 (6)	C29—C30—C31	121.2 (3)
C22—N4—C24	117.6 (3)	C29—C30—H30	119.4
C22—N4—Cu1	131.4 (2)	C31—C30—H30	119.4
C24—N4—Cu1	111.0 (2)	C32—C31—C36	117.2 (3)
C3—C4—C12	116.2 (4)	C32—C31—C30	124.3 (4)
C3—C4—C5	125.7 (4)	C36—C31—C30	118.4 (4)
C12—C4—C5	118.0 (4)	C33—C32—C31	119.8 (4)
Cl6—Cu4—Cl5	175.80 (6)	C33—C32—H32	120.1
C25—N5—C35	118.6 (3)	C31—C32—H32	120.1
C25—N5—Cu2	125.9 (2)	C32—C33—C34	119.3 (4)
C35—N5—Cu2	115.5 (2)	C32—C33—H33	120.4
C6—C5—C4	121.9 (4)	C34—C33—H33	120.4
C6—C5—H5	119.0	N6—C34—C33	122.4 (4)
C4—C5—H5	119.0	N6—C34—H34	118.8
C34—N6—C36	118.4 (3)	C33—C34—H34	118.8
C34—N6—Cu2	131.8 (2)	N5—C35—C28	122.5 (3)
C36—N6—Cu2	109.4 (2)	N5—C35—C36	117.2 (3)
C5—C6—C7	121.4 (4)	C28—C35—C36	120.3 (3)
C5—C6—H6	119.3	N6—C36—C31	122.9 (3)
C7—C6—H6	119.3	N6—C36—C35	117.3 (3)
C37—N7—C47	118.0 (3)	C31—C36—C35	119.8 (3)
C37—N7—Cu2	131.3 (2)	N7—C37—C38	122.0 (3)
C47—N7—Cu2	110.62 (19)	N7—C37—H37	119.0
C8—C7—C11	116.5 (4)	C38—C37—H37	119.0
C8—C7—C6	125.2 (4)	C39—C38—C37	119.9 (3)
C11—C7—C6	118.3 (4)	C39—C38—H38	120.0
C46—N8—C48	118.8 (3)	C37—C38—H38	120.0
C46—N8—Cu2	126.5 (2)	C38—C39—C40	120.1 (3)
C48—N8—Cu2	114.7 (2)	C38—C39—H39	120.0
C9—C8—C7	120.9 (4)	C40—C39—H39	120.0
C9—C8—H8	119.5	C47—C40—C39	116.7 (3)
C7—C8—H8	119.5	C47—C40—C41	118.4 (3)
C8—C9—C10	118.6 (4)	C39—C40—C41	124.9 (3)
C8—C9—H9	120.7	C42—C41—C40	121.5 (3)
C10—C9—H9	120.7	C42—C41—H41	119.3
N2—C10—C9	122.2 (4)	C40—C41—H41	119.3
N2—C10—H10	118.9	C41—C42—C43	121.8 (3)
C9—C10—H10	118.9	C41—C42—H42	119.1
N2—C11—C7	122.7 (4)	C43—C42—H42	119.1
N2—C11—C12	117.4 (3)	C44—C43—C48	116.9 (3)
C7—C11—C12	119.8 (3)	C44—C43—C42	124.9 (3)
N1—C12—C4	122.6 (3)	C48—C43—C42	118.2 (3)
N1—C12—C11	116.9 (3)	C45—C44—C43	120.1 (3)
C4—C12—C11	120.4 (3)	C45—C44—H44	119.9
N3—C13—C14	122.5 (4)	C43—C44—H44	119.9
N3—C13—H13	118.8	C44—C45—C46	119.6 (3)
C14—C13—H13	118.8	C44—C45—H45	120.2
C15—C14—C13	119.5 (4)	C46—C45—H45	120.2
C15—C14—H14	120.3	N8—C46—C45	122.0 (3)
C13—C14—H14	120.3	N8—C46—H46	119.0
C14—C15—C16	119.8 (3)	C45—C46—H46	119.0
C14—C15—H15	120.1	N7—C47—C40	123.3 (3)
C16—C15—H15	120.1	N7—C47—C48	116.6 (3)
C15—C16—C23	117.3 (3)	C40—C47—C48	120.1 (3)
C15—C16—C17	124.6 (3)	N8—C48—C43	122.5 (3)
C23—C16—C17	118.1 (3)	N8—C48—C47	117.4 (3)
C18—C17—C16	121.4 (4)	C43—C48—C47	120.1 (3)
